# Esthesioneuroblastoma: An Unusual Presentation with Intractable Headache

**DOI:** 10.7759/cureus.3653

**Published:** 2018-11-28

**Authors:** Muhammad Noor, Alexander Leyva, John V Dennison, Barbara Manchec, Dhruv Patel

**Affiliations:** 1 Radiology, Florida Hospital-Orlando, Orlando, USA; 2 Radiology, Osceola Regional Medical Center, Kissimmee, USA

**Keywords:** esthesioneuroblastoma, olfactory neuroblastoma, nasal cavity, nasal cavity mass, intractable headache, nasal obstruction, epistaxis, nasal mass, nasal tumor, nasal cavity tumor

## Abstract

Esthesioneuroblastoma, also known as olfactory neuroblastoma, is a malignant tumor of the upper nasal cavity. This report illustrates the case of a 63-year-old woman who presented with intractable headaches. Subsequent radiologic evaluation and correlation with histopathologic analysis confirmed esthesioneuroblastoma. We review herein the typical computed tomographic (CT) and magnetic resonance (MR) imaging findings related to this locally destructive tumor, the prompt diagnosis of which may help prevent long-term morbidity and potentially, mortality. Up-to-date diagnostic criteria, staging, and management considerations are also outlined.

## Introduction

Esthesioneuroblastoma, also known as olfactory neuroblastoma, is a rare and malignant tumor of the upper nasal cavity. The incidence follows a bimodal distribution with peaks at the second and sixth decades of life. Common presenting symptoms include epistaxis and unilateral nasal obstruction; however, the case discussed herein is an unusual presentation involving intractable headaches due to an intracranial extension of the tumor [1-2]. The locally destructive and highly aggressive nature of this tumor necessitates a staging system based on fine anatomic details obtained by computed tomography (CT) and magnetic resonance imaging (MRI) examinations. Although the differential list for a mass involving the nasal cavity is extensive, certain radiographic findings can suggest esthesioneuroblastoma, especially once the mass invades through the cribriform plate into the anterior cranial fossa. Definitive diagnosis is achieved by histopathologic examination. While no stern consensus for treatment exists, management usually consists of surgical resection of the tumor, if possible, followed by post-surgical radiotherapy or chemotherapy [3].

## Case presentation

A 63-year-old woman with a past medical history of hyperlipidemia, diabetes mellitus, and a remote ischemic stroke presented with intractable headaches of five-day duration. Headaches were described as constant, unrelenting, and throbbing in nature. The pain was described as predominantly occipital, which radiated throughout the rest of her head. The month prior to presentation, the patient underwent a rhinoscopy with nasal polypectomy at an outside facility due to a six-month history of progressively worsening unilateral left nasal passage obstruction. The patient was unaware if histopathologic analysis was performed on the removed specimen.

**Figure 1:**
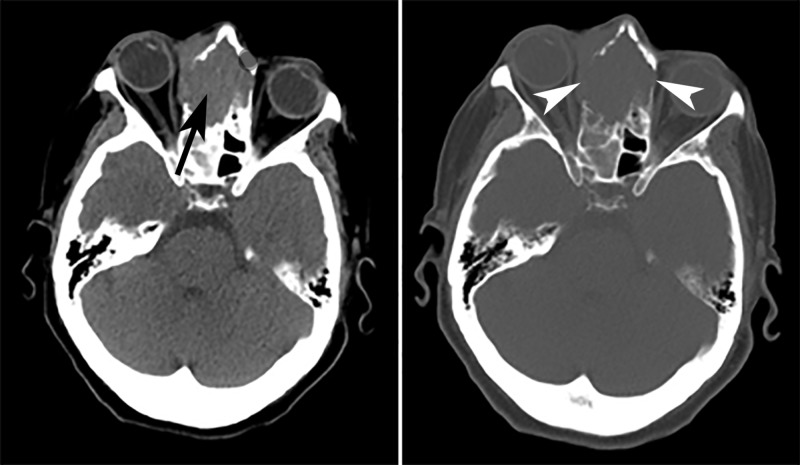
CT head Axial, soft-tissue (*left*) and bone (*right*) windowed CT images of the head demonstrate an expansile soft tissue mass (*arrow) *extending into the bilateral nasal cavity and right orbital cavity with associated osseous destructive change (*arrowheads*) of the medial orbital walls, ethmoid air cells and nasal bone. CT: computed tomography

Upon presentation to the emergency department, the patient underwent a CT evaluation of the head without contrast, which demonstrated a midline aggressive-appearing tumor versus infectious process centered in the bilateral nasal cavities, paranasal sinuses, and right orbit with an intracranial extension to the bilateral frontal lobes. Edema was noted with a mass effect and a midline shift of 4 mm to the left (Figure 1).

**Figure 2:**
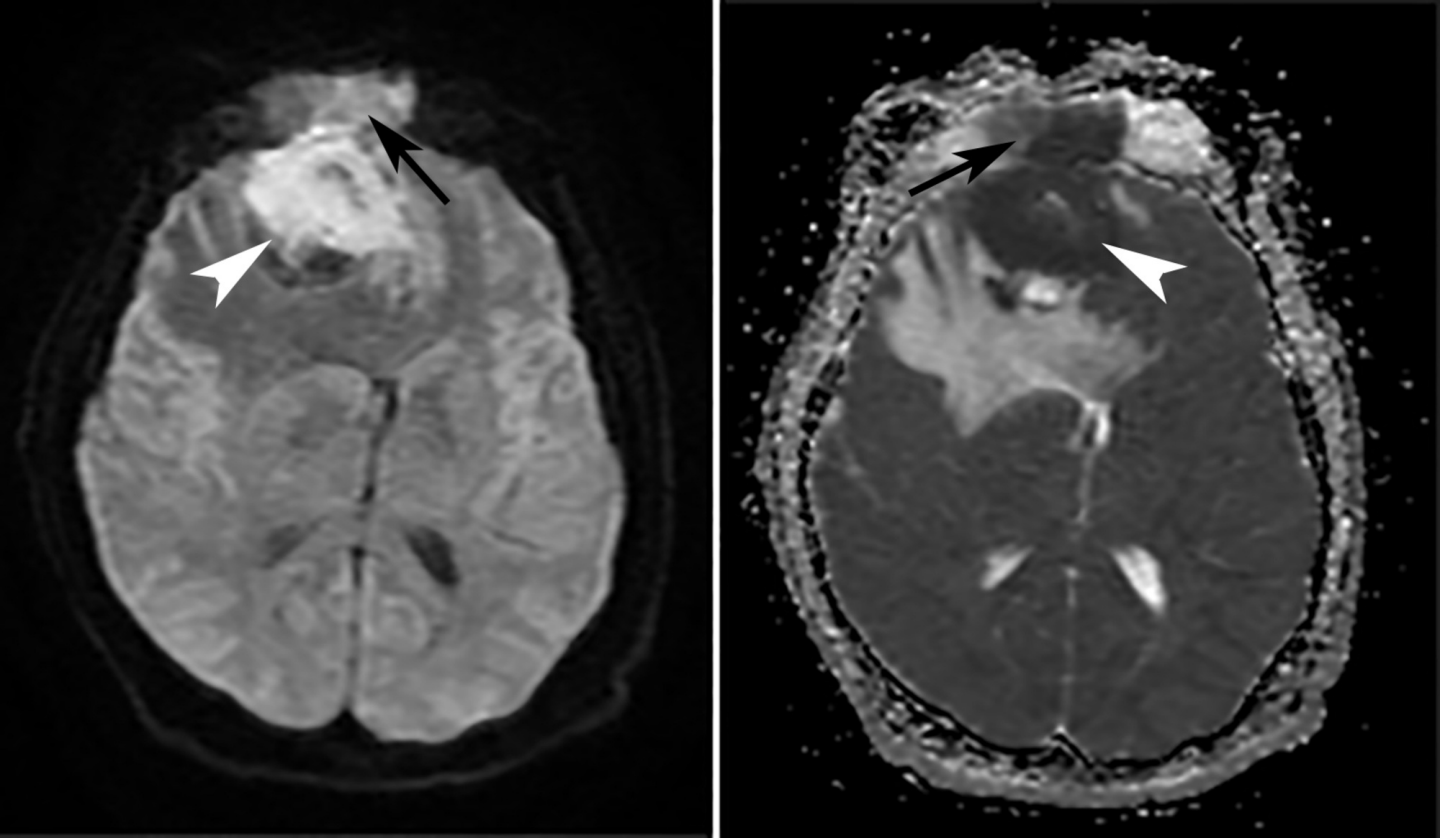
MRI head, DWI, and ADC Axial DWI (*left*) and ADC map (*right*) demonstrate an area of restricted diffusion (dark on DWI, bright on ADC) in a mass (*arrowheads*) involving the medial right frontal lobe and right frontal sinus (*arrows*), a finding suggestive of high cellularity. DWI: diffusion-weighted imaging, ADC: apparent diffusion coefficient

**Figure 3:**
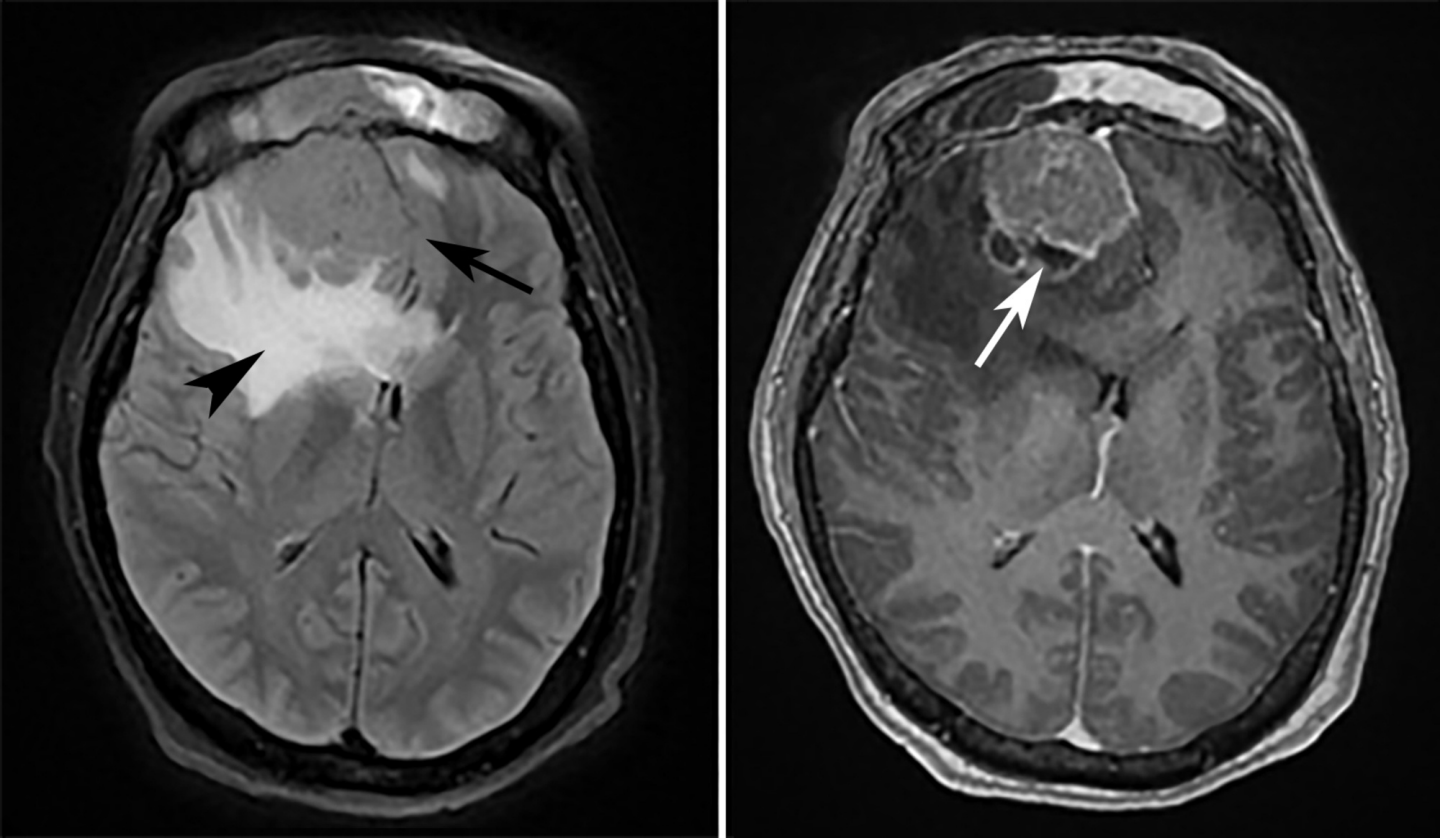
MRI head, T2 FLAIR, and T1 post-contrast Axial T2-weighted FLAIR (*left*) and T1-weighted post-contrast (*right*) MRI images demonstrate a soft tissue mass within the right frontal lobe with mass effect on the medial left frontal lobe (*black arrow*). T2 FLAIR hyperintense signal surrounding the mass (*arrowhead*) is consistent with vasogenic edema. T1 rim and heterogeneous central enhancement is visualized, with an area of hypointense signal at the posterior margin, suggestive of necrosis (*white arrow*). FLAIR: fluid-attenuated inversion recovery, MRI: magnetic resonance imaging

**Figure 4:**
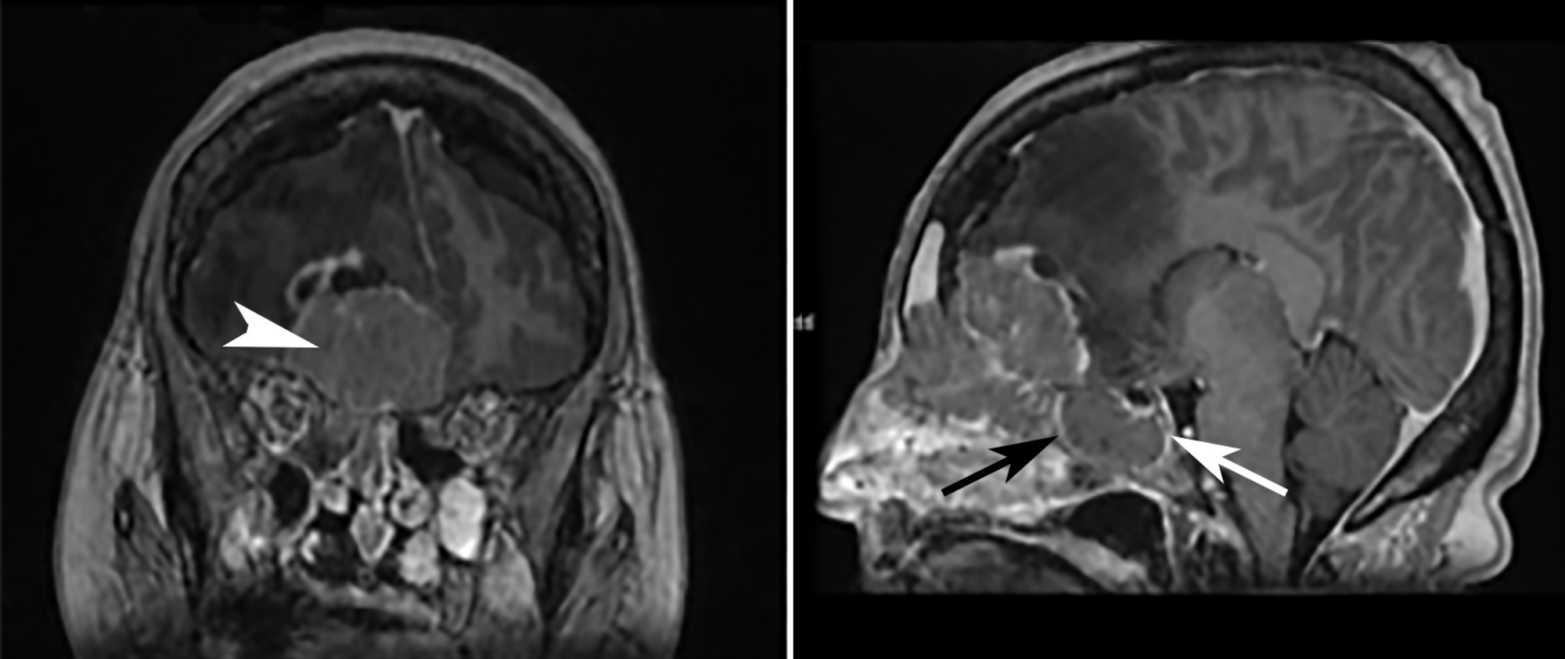
MRI head, T1 coronal and sagittal Coronal (*left*) and sagittal (*right*) T1-weighted post-contrast reformats demonstrate intracranial extent of the mass (*arrowhead*). In addition to the previously noted areas of involvement, rim-enhancing soft tissue signal is also seen involving the sphenoid sinus (*arrows*). MRI: magnetic resonance imaging

Subsequent evaluation with MRI of the head demonstrated a large, hypercellular mass involving the anterior aspect of the right frontal lobe with erosion through the cribriform plate and lamina papyracea, with additional extension into the superior nasal cavity and superomedial right orbit. An extensive vasogenic edema within the right frontal lobe was also visualized, along with a significant mass effect with a 1.1 cm of right-to-left midline shift (Figures 2-4). Pathology later confirmed the radiographic suspicion of esthesioneuroblastoma. The patient began an inpatient course of steroids, and further management was completed by an outpatient neurosurgeon.

## Discussion

Esthesioneuroblastoma, also known as olfactory neuroblastoma, is a malignant tumor of the upper nasal cavity. It is thought to arise from cells in the olfactory epithelium of neural crest cell origin. This cancer exhibits a bimodal age distribution with peaks at the second and sixth decades of life. It accounts for three percent of all intranasal tumors [1]. Over 1200 cases of esthesioneuroblastoma have been reported since the characterization of this malignancy in 1924 [2]. The rarity of this tumor precludes a stern consensus on etiology and management. Patients with this highly invasive mass most commonly present with either unilateral nasal obstruction or epistaxis. Less common presentations occur if the mass has extended beyond the nasal cavity and these include hyposomnia, headache, and vision loss or diplopia if the optic tract is involved [1].

Unfortunately, there are several other aggressive processes that can occur in the upper nasal tract and imaging alone is often unable to definitively distinguish esthesioneuroblastoma from these other processes. However, suggestive findings include unilateral origination of the mass from the superior olfactory recess medial to the middle turbinate, which is often completely opacified, and involvement of the anterior and middle ethmoid air cells. Most commonly, the mass will also be seen invading superiorly through the cribriform plate and into the anterior cranial fossa. This aggressive malignancy usually may cause destructive changes of the surrounding bone depending on the extent of disease [3]. Due to erosion of the cribriform plate and the orbital wall in the patient described here, other considerations included an aggressive meningioma or hemangiopericytoma, as well as a nasopharyngeal carcinoma with an intracranial extension. The complete differential for a mass involving the nasal cavity is extensive and also includes olfactory neuroepithelioma, rhabdomyosarcoma, lymphoma, chordoma, and melanoma metastasis, in addition to many others [4].

Two main classification systems have been employed for the evaluation of esthesioneuroblastoma. The most commonly used system is the modified Kadish classification, which utilizes an A-D scale (Table 1) [5]. The Dulguerov system follows the traditional TNM staging features (tumor location, nodal involvement, and metastatic foci), which allows staging of esthesioneuroblastoma by radiologic evaluation (Table 2) [6].

**Table 1:** Modified Kadish staging system of esthesioneuroblastoma

Modified Kadish – Staging of Esthesioneuroblastoma	
Stage A	Mass confined to the nasal cavity
Stage B	Involvement of nasal cavity and paranasal sinuses
Stage C	Local extension beyond nasal cavity and paranasal sinuses into the surrounding structures
Stage D	Metastasis to cervical lymph nodes and beyond

**Table 2:** Dulguerov staging system of esthesioneuroblastoma

Dulguerov​​​​​​​ – Staging of Esthesioneuroblastoma	
T1	Mass involving nasal cavity +/- paranasal sinuses, excluding sphenoid
T2	Mass involving nasal cavity +/- paranasal sinuses, including sphenoid, with extension to/erosion of cribriform plate
T3	Extension into orbit or anterior cranial fossa. No dural involvement
T4	Tumor involving brain
N0	No nodal involvement
N1	Any form of cervical node involvement
M0	No metastasis
M1	Distant metastasis

For classification purposes, it is important to identify the involvement of the cervical and retropharyngeal nodes. Definitive diagnosis is by histopathological evaluation and involves immunohistochemistry. Prognostic factors and diagnosis depend on Hyam’s I-IV grading system that accounts for various pathological findings [7]. Management consists of surgical resection of the tumor, if possible, followed by post-surgical radiotherapy or chemotherapy. Although recurrence rates are high and a long-term follow-up is required, prognosis and five-year survival rates are favorable with treatment compared with other aggressive tumors of this location. Localized tumors within the Kadish A parameters have a five-year survival rate of about 80% [5].

## Conclusions

The prompt identification and treatment of esthesioneuroblastoma is required for a favorable outcome and depends upon the close collaboration of radiology, pathology, and surgery. Staging, management, and prognosis are in large part determined by regional involvement in non-metastatic cases due to the destructive nature and proximity to neurological structures.
